# Mini review: Interleukin-32 as a key mediator of type 1 diabetes pathogenesis

**DOI:** 10.3389/fimmu.2025.1641698

**Published:** 2025-09-04

**Authors:** James A. Pearson, Stephanie J. Hanna

**Affiliations:** Diabetes Research Group, Division of Infection and Immunity, School of Medicine, Cardiff University, Cardiff, United Kingdom

**Keywords:** IL-32, type 1 diabetes, immunotherapy, T cells, beta cells, β-cells

## Abstract

Type 1 diabetes (T1D) is an autoimmune disease characterized by the destruction of insulin-producing β-cells in the pancreatic islets. The pathogenesis, involving complex interactions between genetic susceptibility and environmental factors, is mediated by T cells driven by multiple stimuli including cytokines. Interleukin-32 (IL-32), a predominantly proinflammatory cytokine, has emerged as a potential contributor to T1D pathogenesis. In this review we discuss current knowledge of IL-32 and its role in T1D pathogenesis, examining expression patterns in PBMCs and islets, possible functional mechanisms, and the potential for IL-32 as a biomarker. We will also consider how immunotherapies currently in clinical trials aiming to slow T1D progression may impact IL-32.

## 1 Introduction

Type 1 diabetes (T1D) is an autoimmune disease characterized by destruction of insulin-producing β-cells in the pancreatic islets by autoantigen-specific T cells. The pathogenesis of T1D involves a complex interaction of genetic risk factors, environmental triggers, and immune dysregulation. T1D is currently treated with exogenous insulin, but in recent years a number of immunotherapies have entered clinical trials with the aim of slowing the loss of β-cells (1). Therefore, it has become crucial to understand how to both target and monitor the immune system in T1D. Among the various inflammatory mediators implicated in T1D, cytokines play a crucial role in orchestrating immune responses and β-cell destruction (2). Whilst many of these cytokines have been well characterized, until recently relatively little was known about IL-32. This review aims to summarize the role of IL-32 in the immune system, with a focus on its impact on T1D progression.

### 1.1 IL-32 structure

Interleukin-32 (IL-32), first identified as natural killer cell transcript 4 (NK4), is a (generally) proinflammatory cytokine that has gained attention for its potential role in various inflammatory and autoimmune diseases, such as rheumatoid arthritis and inflammatory bowel disease (3). IL-32 has 35 known splice variants (https://useast.ensembl.org/Homo_sapiens/Gene/Splice?g=ENSG00000008517;r=16:3065297-3082192) of which 29 produce proteins, varying in length from 131 to 234 amino acids. All of these, except IL-32γ, lack a typical secretory peptide sequence, and indeed bear little structural resemblance to other cytokines (3, 4). Orthologues have been found in primates but not in rodents, whilst in other mammals, putative homologues have very low sequence alignment with human IL-32 (5). The lack of a rodent model has likely contributed to the lack of knowledge of IL-32; however, *in vitro* and *in vivo* human studies are driving our better understanding of IL-32 in both health and disease settings.

### 1.2 Induction of IL-32 expression

IL-32 is highly expressed in CD4^+^ T cell subsets including Tregs, Th1, Th17, Th17.1, Tfh and Th2 cells, as well as activated and memory CD8^+^ T cells, with lower expression in naïve T cells (3, 6). In many cases, the isoforms produced have not been assessed, although the majority of PBMC subsets appear to produce at least the α, β, γ and δ isoforms (reviewed in (3)). Recent work suggests that CD4^+^ T cells predominantly produce IL-32β and are the major source of IL-32β found in the serum (7). IL-32 is also highly expressed in NK cells; however, expression is generally low in both monocytes and naïve B cells (3). IL-32 is often observed as upregulated in disease states, including autoimmunity, in these cells (3, 6, 8, 9).

The expression of IL-32 is induced in T cells, other leukocytes and cells such as epithelial cells and fibroblasts by various proinflammatory cytokines including IL-1β, TNFα, IFNγ, IL-12, IL-18 and IL-23 (reviewed in (3)). In NK cells IL-2 is a strong inducer of IL-32 with a somewhat weaker effect in T cells (10). Recently, it has been shown that IL-32 (particularly IL-32β) is produced in response to IL-2 and secreted via membrane pores and exosomes in response to TCR stimulation (7). In T cells, IL-32 can be induced by *in vitro* stimulation of T cells using anti-CD3 antibodies or PMA and ionomycin (10). This induction by pro-inflammatory cytokines and T cell activation has important implications for the role of IL-32 in T1D.

IL-32 can also be induced in response to hypoxia via HIF1α and cysteamine dioxygenase (ADO) (11). As both IL-1β and IL-18 can induce IL-32, it is not surprising that innate immune receptors such as Toll-like receptors (TLRs) (12, 13) and inflammasomes e.g. NLPR3 (14), which induce IL-1β and IL-18, are associated with increased IL-32 induction. Recent research has drawn attention to both the role of hypoxia (15) and TLR signaling (16) in T1D development, suggesting an additional role for IL-32 in T1D pathogenesis.

### 1.3 IL-32 functions

A classical receptor for IL-32 has not been identified; however it can bind proteinase 3 (PR3), a neutrophil granule serine protease (17), and can bind integrins (αVβ3, αVβ6 but not αVβ8) through an RGD domain (18, 19). Although, as mentioned above, most isoforms lack a secretory domain, IL-32 can be measured in the serum and therefore is likely to function both intra- and extracellularly.

The most widely studied isoforms (α, β, γ, δ), are thought to have distinct biological functions (5) with all four inducing IL-6 production from PBMC, but only the latter three capable of inducing TNFα (20). The isoform-specific actions of IL-32 have been summarized previously (3). All IL-32 isoforms can induce IL-8 production, although IL-32γ and IL-32θ were the most potent (18). IL-32γ is thought to be more proinflammatory, inducing TNFα and IL-6 in rheumatoid arthritis synovial fibroblasts whilst the IL-32β isoform is thought to reduce inflammation (21). Pro-inflammatory cytokines such as TNFα and IFNγ are known to play important roles in T1D development and thus it can be seen that IL-32 may contribute to T1D pathology (1).

Whilst lentiviral knock-down of IL-32 expression reduced CD8^+^ T cell production of IFNγ, it also reduced expression of FoxP3 by Tregs *in vitro* (22). Conversely, *in vitro* culture of PBMCs with IL-32α led to Treg cell death and downregulation of FoxP3 expression (9). These apparently contradictory studies are likely explainable by the role of different IL-32 isoforms acting intracellularly or extracellularly. Therefore, care must therefore be taken when considering monitoring or targeting IL-32 therapeutically in T1D to analyze the various isoforms and assess their differential functions.

IL-32 can induce apoptosis in a wide variety of cell types including T cells (10). Again it is likely that this function is enacted by specific isoforms, as in cell lines IL-32γ and IL-32β, but not IL-32α, induced caspase-8-dependent cell death (23).

IL-32 can aid mitochondrial metabolism (via interactions with the electron transport chain and promotion of oxidative phosphorylation), and promotes proliferation, and differentiation of plasma cells (11) (this effect was driven by intracellular IL-32β and various IL-32 isoforms added to the extracellular media had no effect, highlighting the complexity of studying IL-32). As oxidative phosphorylation is also key in driving activation of T cells and decisions between pathogenic and regulatory T cells (24), the role IL-32 has in this setting should be further investigated.

IL-32, particularly the IL-32γ isoform, has been shown to activate Langerhans cells in the skin and induce CD80, HLA-DR and CXCL10 production (25). This is of particular interest in T1D, where CXCL10 is raised in the peripheral blood (26) and CXCR3 expressed in the islets aids recruitment of autoantigen-specific T cells and thus is a target for immunotherapy (27). In DCs, IL-32γ can upregulate the expression of the chemokines CCL2, CCL4, and CCL5, with upregulation of CCL5 in particular leading to increased chemotaxis of T cells (28). Further, IL-32 can induce monocytes to differentiate into macrophage-like cells (29) and IL-32θ in particular can induce monocytes to differentiate to inflammatory M1 macrophages that produce IL-1β, TNFα and inducible nitric oxide synthase (30). In T1D due to the high numbers of macrophages that infiltrate the islets (31) and their role in driving CD8^+^ T cell destruction of the β-cells (32), IL-32 could therefore be important in modulating these autoimmune responses.

## 2 IL-32 in the serum and its role in other autoimmune and immune system-related diseases

Serum levels of IL-32 are elevated in a number of autoimmune conditions including Graves’ disease (where the percentage of IL-32α+ T cells was also increased) (33). In psoriasis IL-32 expression is increased in infiltrating Tregs, Th cells and cytotoxic T cells, although there are conflicting reports of whether IL-32 is increased in the serum (34, 35). IL-32α is elevated in the serum in myasthenia gravis (36) (reviewed (3)). In ankylosing spondylitis IL-32γ was elevated in the synovial fluid and in rheumatoid arthritis IL-32 was raised in synovial biopsies, correlating with pro-inflammatory cytokine levels and decreased with anti-TNFα therapy (37, 38). *In vitro* experiments suggested this effect was predominantly driven by the IL-32γ isoform. Similarly in Behçet’s disease IL-32 is raised in the serum and in the CSF of neuro-Behcet’s (14, 39). In IBD IL-32α was elevated in the mucosa (40) (reviewed (3)), whilst in atopic dermatitis, serum levels of IL-32 correlated to severity and were reduced with successful treatment (41), potentially implicating IL-32 as a useful biomarker for disease progression and favorable therapy responses.

Serum levels of IL-32 can also be affected by SNPs in the *IL32* gene https://www.ebi.ac.uk/gwas/genes/IL32 (42) and the presence of a C allele or CC genotype in SNP rs45499297(T/C) has been implicated in the elevated serum levels of IL-32 seen in some people with multiple sclerosis and an earlier age of onset (14, 43), as well as a risk factor for the development of multiple sclerosis (44). In people with rheumatoid arthritis the SNP rs4786370 (T/C) CC genotype in the *IL32* promoter was associated with a favorable lipoprotein profile (45) but also with higher IL-32 and pro-inflammatory cytokine production by PBMC (46). People with SLE are reported to have lower levels of IL-32 in the serum and the presence of the *IL32* SNP rs28372698 (A/T) TT genotype was associated with SLE susceptibility (47). SNPs rs10431961(C/T) presence of T allele and rs7188573 (T/C) presence of C allele in the *IL32* region were associated with juvenile idiopathic arthritis risk as well as extent of *IL32* methylation (48). Although in a recent GWAS of T1D *IL32* SNPs were not identified as significantly contributing to overall T1D risk (49), the effects of specific SNPs on age of development, endotype or response to immunotherapies have not been specifically investigated nor have IL-32 levels in the serum been studied in T1D.

## 3 IL-32 expression in PBMC in T1D

Kallionpää et al. performed longitudinal bulk RNAseq on peripheral blood samples from children as they progressed to diabetes and found that high IL-32 expression in CD4^+^, CD8^+^, CD4^−^CD8^−^ cells, and PBMC fractions from peripheral blood was strongly associated with seroconversion and progression to T1D (50). Utilizing scRNAseq the authors identified highly activated and differentiated T cells and NK cells as a major source of IL-32 (50). In a further study, a locus at the promoter of *IL32* was hypomethylated in CD8^+^ T cells of children who progressed to T1D compared to controls. This hypomethylation is thought to increase IL-32 expression (51).

Furthermore, when Honardoost et al. compared the upregulated genes in the PBMC of people living with T1D to healthy controls, using upregulated genes identified by Kallionpää et al. (50) and Fasolino et al. (31), they again identified IL-32 as overexpressed in those with T1D, specifically in the CD4^+^ T cells, CD8^+^ T cells, Tregs, MAIT, VD2^+^ γδT cells and NK cells (52).

It has been demonstrated that *IL32* is highly expressed by CD4^+^ T cells from people living with T1D, as assessed by single cell sequencing, in response to neo- and native epitopes of diabetes autoantigens (53). Furthermore, Okamura et al. incubated PBMCs from people living with T1D with a pool of insulin peptides (mainly 15mers) for 2 hours *in vitro* and found that this stimulation significantly increased the expression of *IL32* in the NK cell subset (54). In contrast, there is also a report of *IL32* downregulation in peripheral blood CD8^+^ T cells in people with T1D compared to controls (55). It should be noted that in these studies, the isoform(s) of IL-32 produced at the protein level have not been determined and as described above these isoforms can activate different pro and anti-inflammatory pathways. Therefore, determination of the isoforms upregulated in T1D should be an urgent priority.

## 4 IL-32 expression in the pancreatic islets in T1D

Fasolino et al. performed scRNAseq of human pancreatic islet cells and in an analysis of differentially expressed genes (DEG) in immune cells between healthy and T1D pancreas samples identified IL-32 as highly differentially expressed (31). In further research, the same group used machine learning to classify whether pancreatic islet cells from autoantibody positive donors were more similar to cells from healthy controls or from people with T1D. They found that IL-32 was a key signature of islet cells from an autoantibody positive donor classified as similar to T1D (56).

Kallionpää et al. demonstrated that exposure to IL-1β and IFNγ induced IL-32 expression in a pancreatic β-cell line (EndoC-βH1), while treatment of the β-cell line with IL-32γ did not affect their survival or ability to produce insulin (50). Similarly, Dettmer et al. also found that stem-cell derived β-cells and the EndoC-βH1 β cell line upregulate IL-32 expression in response to pro-inflammatory cytokines IL-1β, TNFα and IFNγ (57). Finally, in a human IL-32γ-expressing transgenic mouse model, when mice expressed human IL-32γ, streptozotocin-induced diabetes was accelerated (58), highlighting IL-32 as an important biomarker for T1D progression.

## 5 IL-32 up- and downstream of drug targets for T1D

**Figure 1:**
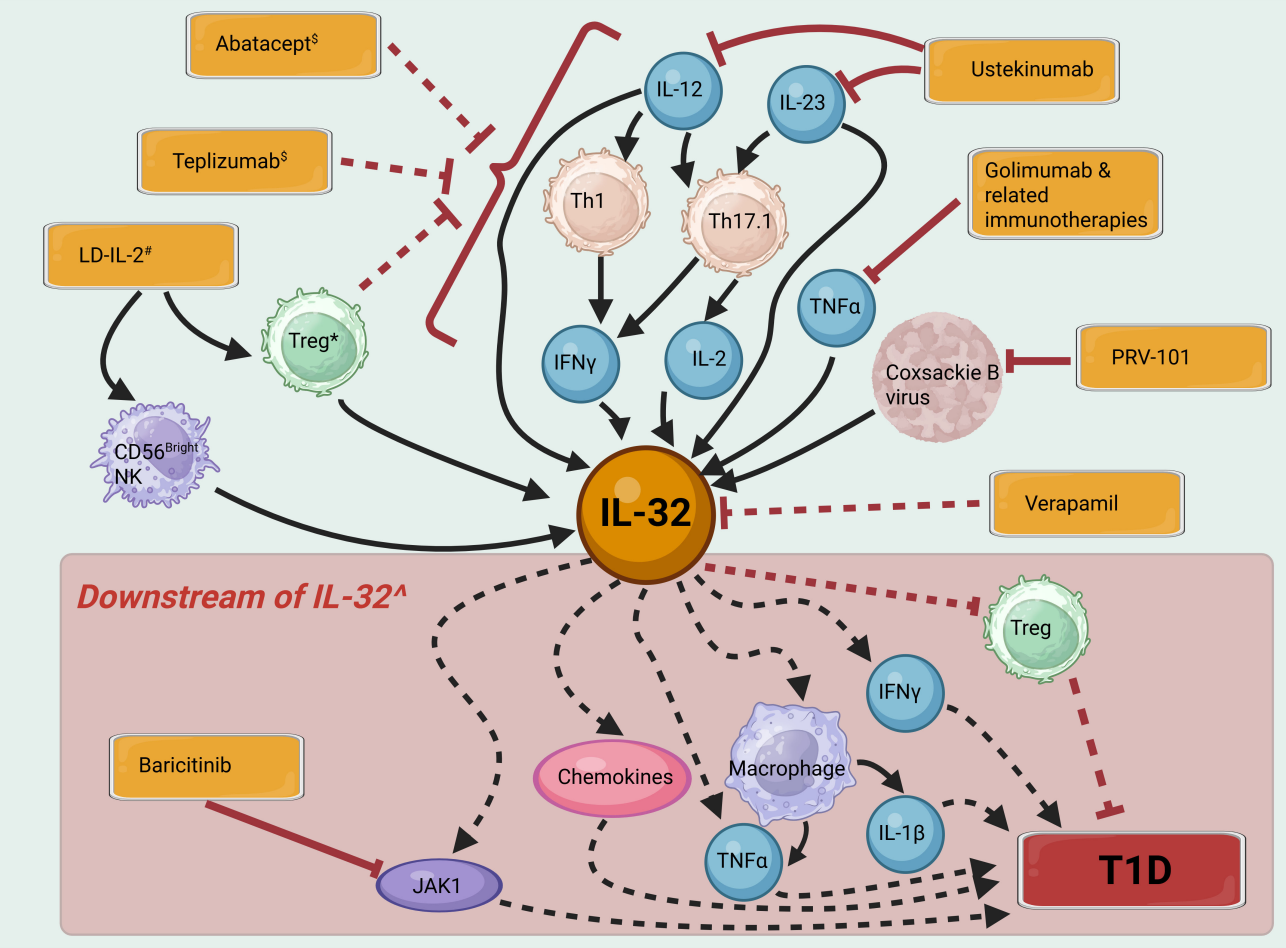
IL-32 signaling pathways and their potential contribution to T1D pathogenesis. Immunotherapies and vaccines are shown in orange boxes. Solid black arrows indicate activation of a pathway, dashed black arrows indirect activation, solid red bars inhibition and dashed red bars indirect inhibition. $ Immunotherapy with Abatacept and Teplizumab aims to induce exhaustion and depletion of a range of effector T cell subsets. # Low dose (LD)-IL-2 may increase IL-32 in some cell subsets, whilst depleting cell subsets producing more proinflammatory isoforms of IL-32. * Whilst Tregs are themselves a source of IL-32, their inhibitory effects on proinflammatory immune cell populations may be expected to have the overall effect of reducing IL-32 levels. ^ Most isoforms of IL-32 are thought to contribute in varying proportions to the pro-inflammatory actions shown here, however IL-32β may have some opposing, anti-inflammatory effects. Created in BioRender. H, S. (2025) https://BioRender.com/5r3h56d.

Taken together, the paragraphs above demonstrate the importance of IL-32 in many of the pathological processes in T1D. Due to the absence of IL-32 in mice, particularly in the well-studied Non-Obese Diabetic (NOD) mouse model of T1D, it remains unknown as to whether in humans, IL-32 function is necessary or sufficient for T1D initiation or progression. Nevertheless, it can be hypothesized that downregulating the expression or function of pro-inflammatory isoforms of IL-32 would be an attractive option in T1D immunotherapy. Yet, as a predominantly intracellular protein with an unclear secretory mechanism, many isoforms, and several putative binding partners but no identified specific receptor (5), it is perhaps unsurprising that no candidate drugs that target IL-32 are in clinical development. However, we can consider its role in specific pathways targeted in recent immunotherapy clinical trials (**Figure 1**, **Table 1**).

**Table 1:** Immunotherapies and vaccines for T1D that influence the IL-32 pathway.

Therapy	Predicted change in IL-32	Cell subsets	Hypothesized mechanism	Ref
Abatacept	↓	Th1, Th17, Th17.1	Decreased abundance and activation of these subsets	(59, 60)
Baricitinib	–	β cells, T cells	Decreased effects of IL-32 via inhibition of JAK/STAT signaling	(61)
Golimumab, other α-TNFα monoclonals	↓	PBMC, DC, lymphoid tissue	Blockade of TNFα-induced IL-32 production	(62)
Low-dose-IL-2	↑?	CD56^bright^ NK cells	Increased expression of IL-32 in NK cells may be offset by increased abundance of FoxP3+Helios+Tregs and decreased IL-21+ CD4+ T cells	(63)
PRV-101	↓	β cells	Prevention of infection of β cells by Coxsackie B virus	(50, 64)
Teplizumab	↓	CD4+ T cells	Exhaustion of cells decreases IL-32 expression	(65)
Ustekinumab	↓	Th17.1, Th1, Th17	Decreased activation of these subsets, reduction in abundance of Th17.1	(66)
Verapamil	↓	β cells (also likely to occur in T cells)	Decreased oxidative stress e.g. through decreased TXNIP expression	(67)

↓, Decreased; ↑, Increased.

### 5.1 Prevention of viral infection

Coxsackie B infection is hypothesized to contribute to T1D development (68) and a vaccine, PRV-101, for coxsackie B is in clinical development with a view to preventing T1D (69). Expression of IL-32 in pancreatic islets is increased by Coxsackie B infection (50). Furthermore, reporter cell lines infected with enterovirus strains isolated from Network for Pancreatic Organ Donors with Diabetes (nPOD) pancreases exhibited increased IL-32 expression compared to those infected with control enterovirus strains (64). Therefore, a potential mechanism of action of the vaccine may be through suppression of IL-32 production and thus should be investigated.

### 5.2 Immunotherapies that alter cytokine signaling

We have recently demonstrated that Ustekinumab, which blocks IL-12 and IL-23 signaling, can slow the loss of insulin production from β-cells in new-onset T1D (66). IL-32 is induced by IL-12 signaling in NK cells and by IL-12, IL-23, IL-2 and IFNγ signaling in T cells ((7) and reviewed (5)). Therefore, Ustekinumab’s blockade of IL-12 and IL-23 signaling, coupled with the downstream decrease in dual IL-17/IFNγ-secreting Th17.1 cells, (particularly those that co-express IL-2), may reduce β-cell loss partly through suppression of IL-32 induction.

TNFα and IL-32 have both been shown to induce each other in a positive feedback loop in arthritis in PBMCs, lymphoid tissue and DCs (62) again suggesting that the effects of Golimumab and other immunotherapies targeting TNFα to slow T1D progression may involve suppression of IL-32 (70, 71).

As discussed above, IL-2 is thought to induce IL-32 expression. However, treatment with low-dose IL-2 is thought to preserve β-cell function primarily through the specific expansion of Treg cells and reduction of IL-21-producing CD4^+^ T cells, without activating effector subsets of T cells and NK cells (63, 72). An examination of IL-32 expression in participants receiving low-dose IL-2 showed a significant upregulation of IL-32 a month after the final dose of IL-2 in CD56^bright^ NK cells after stimulation with PMA and ionomycin (63). This population of IL-32-expressing CD56^bright^ NK cells induced by low-dose IL-2 is thought to have immunoregulatory properties and therefore the impact of increased IL-32 expression is not clear but should be examined in future studies. If the IL-32 isoforms upregulated are predominantly pro-inflammatory (e.g. IL-32γ) this may reduce the immunoregulatory effect of the CD56^bright^ NK cells, whereas if the IL-32 isoforms have anti-inflammatory actions (e.g. IL-32β) this may potentiate the immunoregulatory properties of the CD56^bright^ NK cells (21, 63).

### 5.3 Inhibition of kinases

Inhibition of JAK1 can reverse T1D in NOD mice (73). In humans, the JAK1/2 inhibitor Baricitinib was successful in slowing progression of T1D in a Phase 2 trial [BANDIT (74)], this research is being continued in the recently launched JAKPOT T1D study (NCT05743244) and T1DPlus (ISRCTN45965456). IL-32 has been demonstrated to upregulate JAK1 expression and increase activation of the JAK1 signaling pathway (61), therefore inhibition with Baricitinib may act to reduce the effects of overexpression of IL-32 in T1D.

### 5.4 Modulation of T cell subsets

Teplizumab, an anti-CD3 monoclonal antibody, currently the sole licensed immunotherapy for delaying the onset of T1D in the USA (75) acts via inhibition of CD3 activation during TCR signaling, leading to modification of T cell subset abundance and the partial exhaustion of CD8^+^ T cells (65). In scRNAseq analysis, *IL32* gene expression was significantly downregulated in CD4^+^ T cells from individuals treated with Teplizumab compared to placebo controls at 18 months (65).

Abatacept, a fusion protein of the extracellular domain of CTLA4 fused to the Fc region of IgG1,has been shown to decrease Th17 and Th1 cell abundance and proliferation in in rheumatoid arthritis (59, 60) and its efficacy is linked to baseline levels of Th17.1 cells. Therefore it may reduce IL-32 expression in T1D through decreased IFNγ signaling; however, as Abatacept also decreases abundance of Tregs the overall balance of its effect on IL-32 levels should also be determined (76).

### 5.5 β-cell preservation with verapamil

Verapamil has been shown in the CLVer study to slow C-peptide loss in T1D (77), a result that is being followed up in Vera-T1D [awaiting publication of results (78)]. Verapamil will also be given to all participants in T1DPlus (in addition to immunotherapies in different arms of the trial). In human islet samples *in vitro* treatment with verapamil has been shown to reduce IL-32 expression and it is hypothesized that this is one of the main pathways through which it exerts its protective effects on β-cells (67). Further investigation is needed to assess the effect of verapamil on IL-32 in immune cells.

## 6 Conclusion

In summary there is an increasing body of evidence which positions IL-32 as a key cytokine involved in the autoimmune pathogenesis of T1D. IL-32 upregulation occurs in peripheral blood immune cells early in the disease process and is a feature of the autoantigen-specific T cell response; however, the expression and functions of the different isoforms, particularly IL-32β which may have anti-inflammatory actions, remain poorly defined.

Many T1D immunotherapies are predicted to impact IL-32 production and signaling. Therefore, there is a strong case to develop IL-32 as a biomarker to not only monitor T1D progression but also to evaluate the effectiveness of immunotherapies in clinical trials. Whilst this could be through analysis of IL-32 at the mRNA or protein level in PBMC subsets, levels of IL-32 in the serum of people with T1D should also be assessed. Monitoring IL-32 as a novel biomarker may help identify the immunotherapy that individuals would respond best to, while also ensuring those at greatest risk of developing T1D are more closely monitored or offered disease-modifying therapy.

Because IL-32 is not expressed in most other mammals and is not targeted by any immunotherapies in clinical development, the field lacks definitive evidence that direct inhibition of IL-32 function would be sufficient to prevent either the initiation of β-cell autoimmunity or the progression of T1D in humans. However, development of monoclonal antibodies to target extracellular IL-32 or adaption of siRNA approaches (79) to knockdown intracellular IL-32 expression would allow this question to be addressed in both T1D and other autoimmune diseases where current evidence suggests a crucial role for IL-32.

## Data Availability

Data sharing not applicable to this article as no datasets were generated or analysed during the current study.
